# Racial and social-economic inequalities in systemic chemotherapy use among adult glioblastoma patients following surgery and radiotherapy

**DOI:** 10.1038/s41598-024-68962-y

**Published:** 2024-08-17

**Authors:** Fei Xu, Xin Hua, Mengdi Wang, Weiguo Cao, Shubei Wang, Cheng Xu, Jiayi Chen, Yunsheng Gao, Linlin Chen, Weiqiong Ni

**Affiliations:** Department of Radiation Oncology, Ruijin Hospital, Shanghai Jiaotong University School of Medicine, 197 Ruijin Second Road, Shanghai, 200025 China

**Keywords:** Global health, GBM, Post-surgery, Beam radiotherapy, Systemic chemotherapy, CNS cancer, Health care economics

## Abstract

Not all patients with glioblastoma multiforme (GBM) eligible for systemic chemotherapy after upfront surgery and radiotherapy finally receive it. The information on patients with GBM was retrieved from the surveillance, epidemiology, and end results database. Patients who underwent upfront surgery or biopsy and external beam radiotherapy between 2010 and 2019 were eligible for systemic chemotherapy. The available patient and tumor characteristics were assessed using multivariable logistic regression and chi-squared test. Out of the 16,682 patients eligible, 92.1% underwent systemic chemotherapy. The characteristics linked to the lowest systemic chemotherapy utilization included tumors of the brain stem/cerebellum (*P* = 0.01), former years of diagnosis (*P* = 0.001), ≥ 80 years of age (*P* < 0.001), Hispanic, Non-Hispanic Asian, Pacific Islander, or Black race (*P* < 0.001), non-partnered status (*P* < 0.001), and low median household income (*P* = 0.006). Primary tumor site, year of diagnosis, age, race, partnered status, and median household income correlated with the omission of systemic chemotherapy in GBM in adult patients.

## Introduction

In the United States, primary intracranial tumor accounts for approximately 1.3% of new cancer diagnoses yearly (25,050 cases) and approximately 3.0% of annual cancer-related deaths (18,280 deaths)^1^. Around 48.6% of primary malignant brain tumors are attributed to glioblastoma (GBM), the most prevalent malignant histology^2^. GBM is well known for its dismal poor prognosis with only a 5% 5-year relative survival rate^2^. The prognosis for overall survival has seen a meaningful extension owing to the increased utilization of adjuvant RT and systemic chemotherapy. Post-operative radiation alone leads to a median survival of approximately less than a year, and the addition of adjuvant TMZ systemic chemotherapy to radiation extends survival to 14–16 months^3^. Additional TMZ exposure in the interval between surgery and concomitant RT extends survival to 17.6 months^4^. The standard of care for newly diagnosed GBM is maximum safe resection followed by the Stupp. Regimen-concurrent radiation with oral daily temozolomide (TMZ) and systemic chemotherapy with adjuvant TMZ, 5 days on and 23 days off for 6–12 cycles^3,5,6^. Other systemic chemotherapy includes the nitrosourea-based regimen (procarbazine, lomustine, and vincristine (PCV) or carmustine)^7^, which has worse toxicity than TMZ. The Radiation Therapy Oncology Group (RTOG) 9813 trial reported a similar survival outcome in individuals treated with radiotherapy (RT) with concurrent nitrosourea than concurrent TMZ but a higher rate of discontinuation of the former due to toxicity (79% vs. 40%, *P* < 0.001)^8^.

A part of patients who undergo upfront surgery or biopsy deviate from the suggested method of systemic treatment. As a result, these individuals that could benefit from adjuvant systemic treatment may not receive it. Previous studies have shown that, in China, although physicians have recommended the Stupp. regimen for 89.1% of GBM patients, only 15.8% received treatment conforming to it^9^. The acceptance of systemic chemotherapy is influenced by various factors, including the socioeconomic backgrounds of patients and the cost-effectiveness ratio of the treatment regimen^10^. It is noteworthy that a single academic institution in Lyon, France, observed a correlation between the increased use of TMZ in newly diagnosed GBM and the rise in total treatment costs^11^. Specifically, the group with a mean cost of 71,148 € per patient received TMZ more frequently than that with a mean cost of 54,388€ (71% vs. 39%, *P* < 0.05). However, considering the context of the French healthcare system, which may differ from the US system, particularly in terms of cost structures and insurance coverage. The socioeconomic factors, as well as the accessibility and affordability of treatments like TMZ, may vary greatly between these two systems. While socioeconomic factor is indeed one of the reasons influencing treatment patterns of GBM. Other factors affecting the utilization of systemic chemotherapy remaining unclear and may involve differences in clinical practices, healthcare policies and patient preferences. Therefore, additional research is necessary to identify and rectify any inequities in treatment administration and outcomes.

Through population-based analysis, this research aimed to obtain an understanding of the usage patterns of systemic chemotherapy for GBM in adult patients. The rates of systemic chemotherapy administration were evaluated in individuals who met current evidence-based recommendations for systemic chemotherapy, using data obtained from the National Cancer Institute's Surveillance, Epidemiology, and End Results (SEER) database.

This study examined various factors contributing to the omission of systemic chemotherapy, including primary tumor site, histologic or molecular pathology, year of diagnosis, age, race, partnered status, and household income influence.

## Methodology

### Data source

The information used in this study was retrieved from the SEER database, an authoritative source of population-based cancer statistics retrieved from participating databases across the country, encompassing roughly 34.6% of the US population.

This study utilized the November 2021 submission of the SEER Research Plus Database, 17 Registries, which was linked to the SEER time-dependent county attributes database. The data employed in the analysis contained no privately identifiable health records. For querying the database and extracting the data, the SEER statistical software, SEER*Stat version 8.4.1, was utilized. The data within the SEER program are accessible to the public. This study adhered to the revised Declaration of Helsinki, and therefore, ethical consent was not deemed necessary.

### Data collection and selection

**Figure 1:**
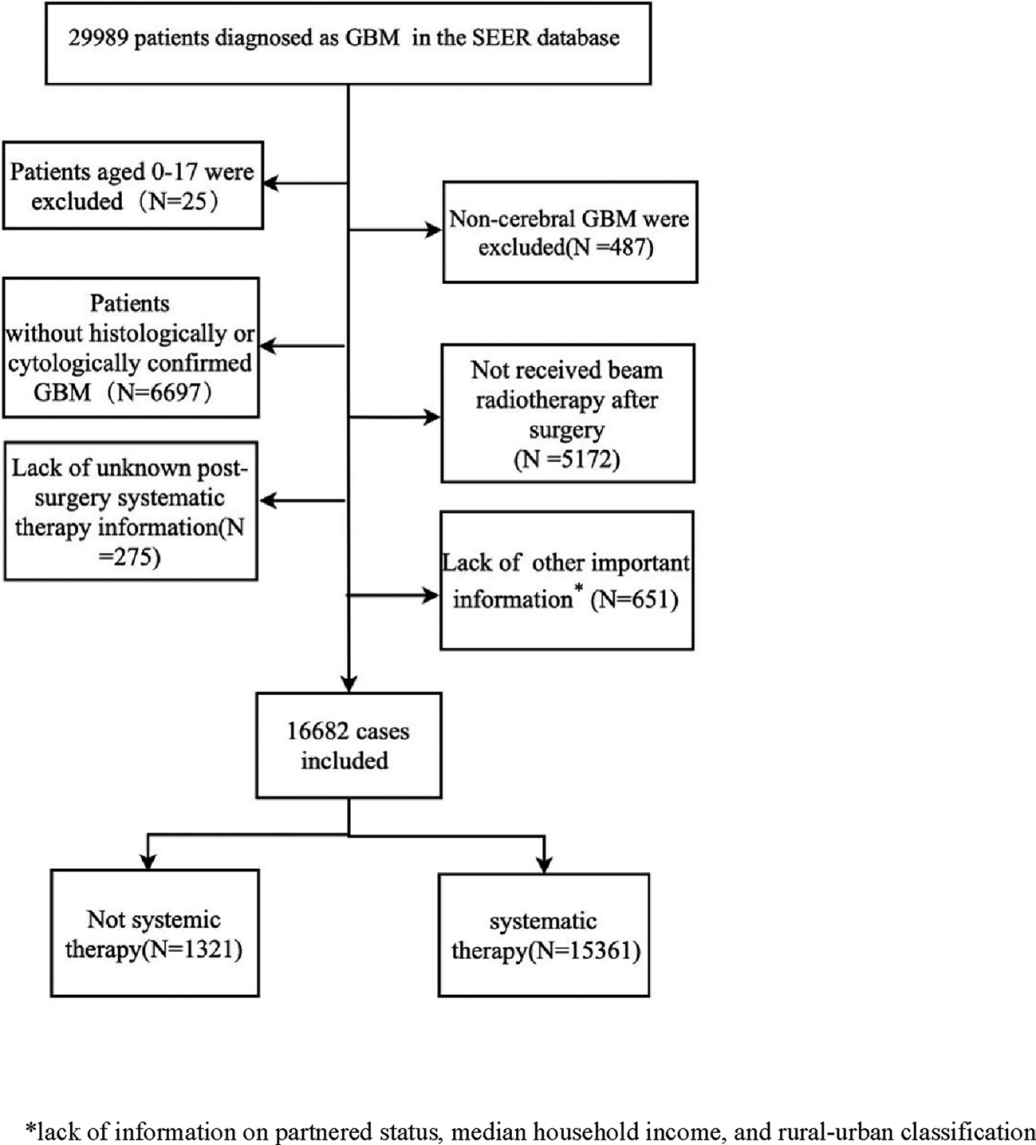
Flow chart indicating the inclusion and exclusion criteria of this study.

The definition of GBM was in accordance with the International Classification of Disease for Oncology; the diagnosis was made using the Adolescents and Young Adults Site Recode 2020 Revision, in which GBM is coded as 3.1.2.2 Glioblastoma–invasive. Pathological tumors were grouped into glioblastoma, NOS (9440/3); glioblastoma, isocitrate dehydrogenase mutant (IDH-mutant) (9445/3); glioblastoma, IDH-wildtype (9440/3); giant cell glioblastoma (9441/3) and gliosarcoma (9442/3). Based on the 2016 World Health Organization Classification of Tumors of the Central Nervous System (2016 CNS WHO classification), IDH-wildtype glioblastoma includes giant cell glioblastoma and gliosarcoma^12^. Figure 1 provides a description of the inclusion and exclusion criteria.

This study included de-identified adult patients newly diagnosed with GBM (age at diagnosis ≥ 18) from the SEER from January 2010 to December 2019. Only the patients with primary tumor sites located in the brain (C71.2-Temporal lobe, C71.3-Parietal lobe, C71.4-Occipital lobe, C71.0-Cerebrum, C71.6-Cerebellum, NOS, C71.7-Brain stem, C71.8-Overlapping lesion of brain, C71.9-Brain, NOS) were included, and those with primary tumor site located in C71.5-Ventricle, NOS, C72.0-Spinal cord, C72.1-Cauda equina, and C72.3-Optic nerve were excluded.

Furthermore, only the patients who underwent cancer-directed surgery or biopsy and with positive histological or cytological diagnostic confirmation were included. Patients with only radiographical or clinical diagnostic confirmation were excluded.

Finally, only patients who underwent beam radiation after surgery were included. Patients with radioactive implants, radioisotopes, radiation methods, or sources not specified who refused radiation and never underwent radiation were excluded.

For the analysis of systemic chemotherapy, the database queries that returned patients with systemic chemotherapy after surgery or no systemic chemotherapy were included, while those with ambiguous information on post-surgery systemic chemotherapy were excluded from the database queries.

The socio-demographic information that was obtained for each patient consisted of their age, race/ethnicity, partnership status at the time of diagnosis, median household income of the county where they lived, and the rural or urban classification of the county of residence, which was determined by the US Department of Agriculture.

### Statistical analysis

Statistical analyses were performed utilizing SPSS 26.0 software (IBM Corp, Armonk, NY). The characteristics of the patients were summarized and compared between those who received adjuvant systemic chemotherapy and those who did not, utilizing the chi-squared test at a significance level of 0.05 (Table 1). To estimate the odds ratios associated with various patient characteristics for receiving adjuvant systemic chemotherapy, a multivariable logistic regression analysis was conducted (Table 2). The multivariable analysis included the following variables: sex, year of diagnosis, age, race, primary site, laterality, 2016 WHO CNS Classification, partnered status, and median household incomes.

**Table 1:** Frequency of adjuvant systemic chemotherapy utilization based on age, race/origin, partnered status, median household income, and rural–urban classification.

	Not received systemic chemotherapy n(%)	Received systemic chemotherapy n(%)	Total	*P*-value
Total	1321(7.9%)	15,361(92.1%)	16,882	
Sex				0.005
Female(40.7%)	586(8.6%)	6199(91.4%)	6785	
Male(59.3%)	735(7.4%)	9162(92.6%)	9897	
Year of diagnosis				< 0.001
2010(8.7%)	130(9%)	1316(91%)	1446	
2011(8.7%)	147(10.2%)	1298(89.8%)	1445	
2012(9.6%)	150(9.4%)	1452(90.6%)	1602	
2013(9.6%)	136(8.5%)	1472(91.5%)	1608	
2014(9.9%)	131(8%)	1514(92%)	1645	
2015(10.1%)	120(7.1%)	1565(92.9%)	1685	
2016(10.4%)	141(8.1%)	1597(91.9%)	1738	
2017(10.8%)	119(6.6%)	1688(93.4%)	1807	
2018(11.1%)	116(6.3%)	1734(93.7%)	1850	
2019(11.1%)	131(7.1%)	1725(92.9%)	1856	
Age				< 0.001
< 50(15.3%)	103(4%)	2443(96%)	2546	
50–59(25.7%)	232(5.4%)	4055(94.6%)	4287	
60–69(33.3%)	338(6.1%)	5213(93.9%)	5551	
70–79(20.8%)	429(12.3%)	3046(87.7%)	3475	
> 80(4.9%)	219(26.6%)	604(73.4%)	823	
Race				< 0.001
Hispanic (All Races)(11.4%)	187(9.9%)	1708(90.1%)	1895	
Non-Hispanic American Indian/Alaska Native(0.3%)	2(3.6%)	53(96.4%)	55	
Non-Hispanic Asian or Pacific Islander(5.1%)	71(8.4%)	778(91.6%)	849	
Non-Hispanic Black(5.2%)	93(10.8%)	772(89.2%)	865	
Non-Hispanic Unknown Race(0.1%)	5(23.8%)	16(76.2%)	21	
Non-Hispanic White(77.9%)	963(7.4%)	12,034(92.6%)	12,997	
Primary site				0.001
Supratentorial(79%)	1014(7.6%)	12,323(92.4%)	13,337	
Frontal lobe(30.2%)	365(7.3%)	4667(92.7%)	5032	
Temporal lobe(29%)	368(7.6%)	4465(92.4%)	4833	
Parietal lobe(16.4%)	229(8.4%)	2504(91.6%)	2733	
Occipital lobe(4.4%)	52(7%)	687(93%)	739	
Cerebellum(0.7%)	20(16.8%)	99(83.2%)	119	
Brain stem(0.1%)	3(12.5%)	21(87.5%)	24	
Others(19.2%)	284(8.9%)	2918(91.1%)	3202	
Laterality				< 0.001
Left-origin of primary(43.8%)	530(7.3%)	6777(92.7%)	7307	
Right-origin of primary(48.6%)	656(8.1%)	7449(91.9%)	8105	
Bilateral(1.4%)	22(9.7%)	204(90.3%)	226	
Not a paired site(6.3%)	113(10.8%)	931(89.2%)	1044	
2016 WHO CNS Classification				< 0.001
Glioblastoma, NOS(77.2%)	1070(8.3%)	11,802(91.7%)	12,872	
Glioblastoma, IDH-mutant(0.7%)	1(0.9%)	111(99.1%)	112	
Glioblastoma, IDH-wildtype(22.2%)	250(6.8%)	3448(93.2%)	3698	
Giant cell glioblastoma(1%)	16(9.8%)	148(90.2%)	164	
Gliosarcoma(2.8%)	46(9.7%)	427(90.3%)	473	
Partnered status				< 0.001
Not Partnered(30.4%)	519(10.2%)	4546(89.8%)	5065	
Partnered(69.6%)	802(6.9%)	10,815(93.1%)	11,617	
Median household income				< 0.001
< $35,000(1.5%)	21(8.5%)	225(91.5%)	246	
$35,000–$39,999(2%)	33(9.8%)	304(90.2%)	337	
$40,000–$44,999(3.7%)	61(10%)	551(90%)	612	
$45,000–$49,999(4.7%)	72(9.1%)	719(90.9%)	791	
$50,000–$54,999(8.1%)	91(6.7%)	1258(93.3%)	1349	
$55,000–$59,999(6.5%)	100(9.2%)	986(90.8%)	1086	
$60,000–$64,999(14.5%)	222(9.2%)	2195(90.8%)	2417	
$65,000–$69,999(15.3%)	191(7.5%)	2361(92.5%)	2552	
$70,000–$74,999(8.1%)	115(8.5%)	1233(91.5%)	1348	
$75,000 + (35.6%)	415(7%)	5529(93%)	5944	
Rural–urban classification				0.794
Counties in metropolitan areas ge 1 million(60.4%)	801(8%)	9272(92%)	10,073	
Counties in metropolitan areas of 250,000 to 1 million(21.5%)	279(7.8%)	3307(92.2%)	3586	
Counties in metropolitan areas of lt 250 thousand(7.1%)	94(7.9%)	1096(92.1%)	1190	
Nonmetropolitan counties adjacent to a metropolitan area(6.5%)	94(8.7%)	990(91.3%)	1084	
Nonmetropolitan counties not adjacent to a metropolitan area(4.5%)	53(7.1%)	696(92.9%)	749

**Table 2:** Multivariate analysis showing association of likelihood of undergoing systemic chemotherapy.

	Odds ratio	95% CI		*P*-value
Sex				
Female	0.96	0.85	1.08	0.446
Male	1			
Year of diagnosis	1.04	1.02	1.07	0.001
Age				< 0.001
< 50	10.16	7.85	13.16	< 0.001
50–59	6.78	5.51	8.35	< 0.001
60–69	5.69	4.69	6.9	< 0.001
70–79	2.51	2.08	3.03	< 0.001
> 80	1			
Race				< 0.001
Hispanic (All Races)	0.61	0.52	0.73	< 0.001
Non-Hispanic American Indian/Alaska Native	2.05	0.49	8.55	0.326
Non-Hispanic Asian or Pacific Islander	0.73	0.56	0.95	0.019
Non-Hispanic Black	0.61	0.48	0.77	< 0.001
Non-Hispanic Unknown Race	0.16	0.06	0.46	0.001
Non-Hispanic White	1			
Primary site				0.01
Others	2.25	1.38	3.65	0.001
Supratentorial GBM	2.34	1.45	3.77	0.001
Frontal lobe	2.40	1.46	3.93	0.001
Temporal lobe	2.45	1.49	4.02	< 0.001
Parietal lobe	2.23	1.35	3.69	0.002
Occipital lobe	2.88	1.64	5.06	< 0.001
Brain stem/Cerebellum	1			
Laterality				0.071
Left—origin of primary	1.10	0.97	1.24	0.13
Bilateral	0.75	0.47	1.19	0.226
Not a paired site	0.83	0.64	1.07	0.146
Right—origin of primary	1			
2016 WHO CNS Classification			0.091	
Glioblastoma, NOS	0.98	0.82	1.17	0.798
Glioblastoma, IDH-mutant	5.15	0.71	37.33	0.105
Glioblastoma, IDH-wildtype	1			
Partnered status				
Not Partnered	0.67	0.59	0.76	< 0.001
Partnered	1			
Median household income	1.03	1.01	1.06	0.006

### Ethical approval

The data within the SEER program are accessible to the public. This study adhered to the revised Declaration of Helsinki, and therefore, ethical consent was not deemed necessary.

## Results

The final patient cohort for statistical analysis consisted of 16,682 individuals identified through the database query. Of these, 92.1% underwent adjuvant systemic chemotherapy, whereas 7.9% did not. The most common subsites were supratentorial GBMs situated in the frontal lobe (30.2%), temporal lobe (29%), parietal lobe (16.4%), and occipital lobe (4.4%). And 19.2% of the supratentorial GBMs were at other sites in the brain, including the overlapping lesions of the brain and brain, Not Otherwise Specified (NOS). The rare sites were the cerebellum (0.7%) and brain stem (0.1%). The common tumor pathologies were glioblastoma, NOS (77.2%), and glioblastoma, IDH-wildtype (22.2%). Glioblastoma, IDH-mutant (0.7%), was not a common subtype. Table 1 presents the summary of the demographic and tumor characteristics of individuals and their use of adjuvant systemic chemotherapy. Table 2 displays the odds ratios for each variable: sex, year of diagnosis, age, race, primary site, laterality, tumor pathology in 2016 WHO CNS Classification, partnered status, and household incomes.

Statistically significant differences in adjuvant systemic chemotherapy utilization were recorded in some of the independent variables. Patients with the primary tumor site cerebellum and brain stem had the lowest likelihood of undergoing systemic chemotherapy (83.2% and 87.5%, respectively) (Table 1). Patients with the primary tumor in all other sites, particularly in the occipital lobe (93%), frontal lobe (92.7%), temporal lobe (92.4%), parietal lobe (91.6%) and other sites in the brain (91.1%), were more likely to undergo systemic chemotherapy (Table 1). Patients with supratentorial GBM had approximately 2.3 times (OR: 2.34, 95%CI 1.45–3.77, *P* = 0.001) odds of undergoing systemic chemotherapy than cerebellar and brain stem, respectively (Table 2). There was no difference in adjuvant chemotherapy use among patients with variable tumor pathologies based on the 2016 WHO CNS Classification or with any laterality (Table 2).

Female individuals had a lower probability of undergoing adjuvant chemotherapy than male individuals (Table 1). Nevertheless, these differences were not statistically significant (Table 2). In terms of the year of diagnosis, the adjuvant chemotherapy uptake increased slightly with each year (OR 1.04, CI 1.02–1.07, *P* = 0.001) (Table 2). Systemic chemotherapy uptake decreased in general as age increased. Patients under 50 had the highest rates of adjuvant chemotherapy utilization (96%), followed by those aged 50–59 (94.6%), respectively. These patients underwent systemic chemotherapy (Table 1). Moreover, 93.9% of patients aged 60–69 and 87.7% aged 70–79 underwent chemotherapy (Table 1). Patients in younger age groups had a greater likelihood of undergoing adjuvant chemotherapy than those aged 80 and above, and these differences reached the significance level (Table 2).

Non-partnered individuals (divorced/separated/single/widowed) had approximately 33% lower odds of undergoing chemotherapy than those who were partnered (OR 0.67, CI 0.59–0.76, *P* < 0.001) (Table 2). Furthermore, significant differences in the odds of undergoing chemotherapy were noted on the basis of the median household income in individuals. The rates of adjuvant chemotherapy utilization increased slightly with increasing household income (OR 1.03, CI 1.01–1.06, *P* = 0.006) (Table 2).

With regard to race/ethnicity, non-Hispanic Blacks, Asians, Pacific Islanders, or Hispanics exhibited a decreased probability of undergoing adjuvant systemic chemotherapy in comparison to non-Hispanic White individuals (Table 1). It was found to be statistically significant for Hispanics (All Races) (OR 0.61, CI 0.52–0.73, *P* < 0.001), Non-Hispanic Asians or Pacific Islanders (OR 0.73, CI 0.56–0.95, *P* = 0.019), and Non-Hispanic Black individuals (OR 0.61, CI 0.48–0.77, *P* < 0.001) (Table 2).

## Discussion

This research included individuals who had undergone surgery or biopsy and received post-surgery beam radiotherapy. The eligibility for adjuvant chemotherapy was determined on the basis of the available tumor and patient characteristics in the SEER database. However, around 7.9% of these individuals did not receive it. The findings of this research highlighted that primary tumor site, year of diagnosis, age, race, partnered status, and household income significantly correlate. In contrast, tumor pathology based on the 2016 WHO CNS Classification and laterality does not considerably correlate with the utilization of chemotherapy in GBM in adult patients (Table 2).

### Tumor factors

The 2021 WHO Classification of Tumors of the CNS recognizes brainstem high-grade glioma as a diffuse midline glioma with H3 K27 alteration, typically affecting children and young adults^13^. Despite treatment with systemic therapy plus radiation, survival remains poor with a median of 10–11 months^14,15^, and systemic therapy(such as TMZ, other chemotherapy agents, or anti-EGFR antibody) may not improve outcomes but increase toxicity^16–19^. Therefore, based on the current results, the addition of systemic therapy is not actively recommended. Novel treatment modalities are needed to be investigated^20,21^. Cerebellar GBM, a rare brain cancer comprising just 0.24–1.00% of all GBM cases (0.24–1.00%)^22,23^ exhibits distinct genomic characterization and biological behaviors that, unlike supratentorial GBM, are not yet fully understood^24,25^. Previous research based on the National Cancer Database has also revealed that cerebellar GBM were less likely to receive chemotherapy (57.4% vs. 64.3%, *P* value < 0.001)^26^. In this study, patients with supratentorial GBM were more likely to receive systemic chemotherapy compared to those with cerebellar or brainstem GBM(OR: 2.34, 95%CI 1.45–3.77, *P* = 0.001). These findings suggest that the treatment strategies for cerebellar GBM or brainstem GBM may be influenced by its unique biological properties, which could potentially affect the response to chemotherapy. New treatment strategies are needed to be investigated to provide greater therapeutic benefits for these patients.

### Patient factors

The administration of adjuvant chemotherapy for GBM can result in adverse events such as hematologic toxicity, neutropenia, and fatigue, which may impact the quality of life of individuals. Therefore, it is crucial to consider these adverse effects against the risk of disease recurrence and the resulting significant morbidity^3^. Consequently, decisions regarding systemic chemotherapy are influenced by various factors, such as life expectancy, age, performance status, comorbidities, and the presence of O6-methylguanine-DNA-methyltransferase (MGMT) promoter methylation^27–29^. The findings of this study indicate a tendency towards reduced chemotherapy utilization in elderly individuals, specifically in those over 80 years old. Patients under 50 have nearly ten times higher odds of receiving chemotherapy than those over 80 (OR 10.16, CL 7.85–13.16, *P* < 0.001). This is in line with literature that investigates the utilization of chemotherapy in elderly populations. The 2009 long-term 5-year follow-up subgroup analyses of the European Organisation for Research and Treatment of Cancer (EORTC) 26,981-National Cancer Institute of Canada (NCIC) CE.3 trial found that combination chemoradiation therapy conferred a survival advantage compared to radiotherapy alone. Nevertheless, this advantage was observed to decline with age, with individuals over 60 years of age experiencing less overall benefit than those receiving only RT (10.9 months vs. 11.8 months)^30,31^. Elderly populations with poor performance status might be unable to endure combined chemoradiation therapy. The 2012 Nordic trials found that hypofractionated RT (hRT) along with the omission of TMZ was superior to standard RT (sRT) for patients > 70 years of age (hRT *vs.* sRT HR: 0.59 (95% CI 0.37–0.93), *P* < 0.0001)^27^. For these patients, the omission of TMZ from hRT may be warranted. However, the 2017 Canadian Cancer Trials Group (CCTG)/EORTC 26,062 trial observed that combinatorial hRT and TMZ decreases the hazard ratio for death by 33% (HR: 0.67; 95% CI 0.56–0.80; *P* < 0.001) than hRT alone^32^ and can be a viable option for elderly GBM with good performance status. Subgroup analyses also reported more survival advantage in patients with O6-methylguanine-DNA methyltransferase promoter methylation (m*MGMT*) treated with combinatorial hRT and TMZ compared with hRT alone (HR: 0.53; 95% CI 0.38–0.73; *P* < 0.001). Based on the evidence from these prospective studies, the treatment regimen needs to be tailored with the consideration of performance status and the presence of m*MGMT* for elderly GBM. Unfortunately, the SEER database does not currently provide information on individual performance status or MGMT status.

Numerous studies have well-documented the influence of partnered status on treatment patterns and outcomes^33–38^. A previous study found that married patients with GBM had a median overall survival advantage (married vs. unmarried: 10 vs. 7 months, *P* < 0.001). However, fundamental mechanisms responsible for this impact are not yet entirely comprehended^39^. This may be attributed to the provision of economic and mental support from partners, which enables the patients to receive an improved quality of treatment. This research confirmed that unpartnered individuals had a lower probability of undergoing adjuvant systemic chemotherapy than partnered individuals (OR 0.67, CI 0.59–0.76, *P* < 0.001). The findings of the study provide additional evidence to support the significant influence of partner support on receiving definitive treatment, such as adjuvant chemotherapy, which may be due to the particularly arduous nature of the treatment. Thus, providing additional support resources for non-partnered individuals may aid in narrowing this disparity.

The role of race as a significant factor contributing to disparities in cancer treatment patterns and outcomes has been extensively studied. Black non-Hispanics and Hispanics had a lower probability of undergoing radiotherapy and chemotherapy than non-Hispanic white patients^40^. Taking into account these well-established disparities, it is not surprising that this analysis determined trends indicating that Black, Hispanic, Non-Hispanic Asian, or Pacific Islander individuals had a lower probability of undergoing systemic chemotherapy than other individuals.

Previous research indicates that the addition of TMZ to GBM treatment raises the average cost from $35,017(surgery + XRT) to $82,018(surgery + XRT + TMZ) , highlighting the need for financial support^41^. In our research, an increase in household income was associated with a statistically significant rise in the likelihood of receiving systemic chemotherapy, underscoring the influence of economic support on treatment pattern decisions. The observed disparities in systemic chemotherapy use likely stem from the U.S. insurance-based healthcare system. The significant variables related to the wealth of the patient/household underscore the role of economic factors in accessing and receiving treatment patterns. It is plausible that insurance coverage, out-of-pocket costs, and the financial burden on patients and their families can significantly affect the decision-making process regarding chemotherapy treatment. This study also found that adjuvant systemic chemotherapy uptake grows slightly year by year^11^. This is concomitant with the report from a single academic institution in Lyon, France, that showed that patients received TMZ more frequently in 2008 than in 2004 (71% vs. 39%, *P* < 0.05). It is understandable that introducing a new treatment approach and gaining patient acceptance may require time.

There were no significant differences in receiving adjuvant systemic chemotherapy based on tumor laterality or pathology by the 2016 WHO CNS Classification. The 2021 WHO CNS Classification recently stratified most IDH-mutant GBM into astrocytoma, WHO grade 4^13^. Numerous studies have shown that the presence of IDH1 mutation serves as a predictive biomarker for TMZ sensitivity in low-grade gliomas and secondary GBM^42,43^. However, the complete IDH status details of 77.2% of the population were unknown.

## Limitations

Due to the nature of the population-based analysis, access to all factors involved in clinical decision-making, such as detailed radiology or pathology reports that indicate tumor size and extent of resection, was unavailable. The SEER database did not contain information on critical factors such as performance status and MGMT status in GBM, which could affect treatment decisions. We hope that future research endeavors prioritize the integration of comprehensive molecular data, such as MGMT status, into analyses. Patient comorbidities and risk factors were not available to us. Specific systemic chemotherapy drugs or the dose or cycles were unavailable to us. In addition, the use of hRT or concurrent chemotherapy was not within the scope of this study and may warrant further investigation in the future.

Although there are limitations to the SEER database, it remains a valuable resource for examining treatment patterns at the population level and generating hypotheses and opportunities for further investigation. This research successfully identified various factors that are significantly associated with the use of systemic adjuvant chemotherapy among GBM individuals.

## Conclusion

In conclusion, it was found that roughly 7.9% of individuals with GBM in adult patients do not undergo adjuvant chemotherapy after surgery as recommended by current evidence-based guidelines. Patients with certain subsite cancers, such as brain stem/cerebellar tumors, as well as those diagnosed in earlier years, aged ≥ 80, of Hispanic, Non-Hispanic Asian, Pacific Islander, or Black race, non-partnered, and with lower median household income, are less likely to undergo adjuvant chemotherapy. Laterality or tumor pathology is not significantly correlated with adjuvant chemotherapy uptake. Furthermore, it was found that all treatment decisions are tailored to the individual patient, and crucial patient-specific data are not assessable at the population level. Additional research is warranted to determine which kind of GBM in adult patients properly omits adjuvant chemotherapy after surgery and adjuvant RT.

## Data Availability

The data that support the findings of this study are openly available in the SEER Research Plus Database, 17 Registries, November 2021 submission, linked to County Attributes—Time Dependent (1990–2019) Income/Rurality, publicly available as detailed at https://www.seer.cancer.gov.
